# MicroRNA-26b-5p suppresses the proliferation of tongue squamous cell carcinoma via targeting proline rich 11 (PRR11)

**DOI:** 10.1080/21655979.2021.1969832

**Published:** 2021-09-07

**Authors:** Liang Yi, Ying Liu, Anji Xu, Sha Li, Hailin Zhang, Mingjing Peng, Zan Li, Huayi Ren, Jie Dai, Chenhui Luo, Yazhou Xiao, Xiao Zhou, Ying Long

**Affiliations:** a Translational Medicine Centre; b Department of Head & Neck Surgery; c Hunan Provincial Clinical Research Centre for Oncoplastic Surgery, Hunan Cancer Hospital and the Affiliated Cancer Hospital of Xiangya School of Medicine, Central South University, Changsha, Hunan, P. R. China; d Hunan Traditional Chinese Medical College, Zhuzhou, Hunan, P. R. China

**Keywords:** Mir-26b-5p, prr11, cell cycle, tongue squamous cell carcinoma, proliferation

## Abstract

MicroRNAs (miRNAs) have been proved to be involved in many biological processes during tumorigenesis and progression, including cell proliferation and cell cycle progression. However, the potential role of miR-26b-5p in tongue squamous cell carcinoma (TSCC) remains unclear. In the present study, we demonstrated that miR-26b-5p was decreased in TSCC tissues in both TCGA-TSCC subset and eight paired samples from TSCC patients, while Proline Rich 11 (PRR11) was obviously increased. Transfection of miR-26b-5p mimics inhibited CALL7 cell proliferation by arresting the cells at the S/G2 transition. Meanwhile, miR-26b-5p inhibitor had the opposite biological functions. The results of luciferase activity and RNA-pulldown assays indicated that miR-26b-5p directly targeted the PRR11 3ʹ -untranslated region in CAL27 cells. Furthermore, the effects of miR-26b-5p on cell cycle regulation were reversed after treatment with siRNA against PRR11. In summary, our findings indicate that miR-26b-5p induce cell cycle arrest in TSCC by targeting PRR11. Hence, targeting miR-26b-5p could be a promising therapeutic strategy for the treatment of TSCC.

## Introduction

Tongue squamous cell carcinoma (TSCC) is the most prevalent malignancy of the oral cavity worldwide [1–3]. Despite advances in detection and treatment, the outcome of patients with TSCC remains unsatisfactory in recent years [4]. In normal cells, cellular growth and proliferation are stringently regulated, while derangements of the cell cycle can lead to uncontrolled proliferation and provide tumor cells with growth advantages. Hence, it is essential to dissect the molecular mechanism supporting the growth advantage of TSCC cells, which might contribute to providing novel therapeutic targets for TSCC treatment.

Accumulating evidence supports the involvement of microRNAs (miRNAs) in the regulation of cell proliferation, apoptosis, invasion, migration and other phenotypes by binding to the 3ʹ- untranslated region (UTR) of its target genes. Previous studies have demonstrated that miR-26b-5p plays important roles in the development and progression of various cancers, including lung cancer, liver cancer and myeloma [5–7]. For instance, miR-26b-5p inhibites cell proliferation and induces apoptosis in multiple myeloma cells by targeting JAG1, and maintains the stemness of hepatocellular carcinoma cells by inhibiting HSC71/HSPA8. miR-26b-5p inhibition promoted the growth of Burkitt lymphoma cells by repressing the KPNA2 expression [8]. Additionally, miR-26b-5p upregulation restricted the malignant features of human apillary thyroid cancer by degrading beta-catenin [9]. To data, The involvement of miR-26b-5p in TSCC remain unclear.

Recent high-throughput studies have facilitated an integrative understanding of the molecular mechanisms underlying carcinogenesis, metastasis, and chemoresistance in cancer research [10–13]. Using bioinformatics tools, we were able to understand miRNA functions by identifying miRNA targets. Proline-rich 11 (PRR11) protein, which has been implicated in the regulation of cell cycle progression [14], was predicted as a candidate target of miR-26b-5p in the current study. Several studies have indicated that PRR11 is overexpressed in various cancers, including lung, ovarian, esophageal and pancreatic cancers [15–18]. However, the potential roles of PRR11 in TSCC remain unclear.

In the present study, miR-26b-5p was found to be downregulated in TSCC, and contributed to the inhibition of cell proliferation. Together with the prediction of the miR-26b-5p binding site within the PRR11 3ʹ-UTR, we hypothesized that miR-26b-5p could suppress TSCC cell proliferation via targeting PRR11. Our results shed new light on the mechanism that provides TSCC cells with growth advantages.

## Materials and methods

### Tissues and cell line

Eight pairs of frozen samples were collected from TSCC patients, and informed consent was obtained from the Hunan Cancer Hospital. All experiments were approved by the ethics committee of the Hunan Cancer Hospital (KYJJ-2020-222), Changsha, China. The TSCC cell line CAL27 was purchased from Shanghai Genechem Co., Ltd, and routinely cultured in DMEM with 10% FBS (Gibco, Gaithersburg, MD).

### Bioinformatics and statistical analysis

The gene expression data (FPKM) of TSCC patients were downloaded and filtered from The Cancer Genome Atlas (TCGA; cancergenome.nih.gov), and the candidate gene data were subsequently extracted to form a new matrix. Differences in gene expression between groups were assessed using the Student’s t-test. Using ENCORI [19], candidate miRNA-target pairs were selected, and the Pearson correlation coefficient (PCC) values between the expressions of two genes in each pair were subsequently calculated. The pairs with PCC< −0.2 and corrected *p*-value < 0.05 were considered statistically significantly correlated, and the correlation between candidate genes in different datasets was visualized as scatter diagrams. Because TSCC is always considered as a subset of head and neck squamous cell carcinoma, overall survival (OS) analysis of candidate genes was performed using the web tool OncoLnc (http://www.oncolnc.org) in the TCGA-HNSC dataset [20]. The Kaplan–Meier method was used to estimate OS.

### Quantitative real-time PCR

Each siRNA, miRNA mimic or inhibitor (GenePharm, Shanghai, China) was transfected into CAL27 cells for 48 h using Lipofectamine 2000 (Invitrogen, Carlsbad, CA). Total RNA was extracted using the Trizol reagent (Invitrogen, Carlsbad, CA), and then reverse transcribed to cDNA using PrimeScriptTM RT-PCR Kit (Takara, Dalian, China) according to the manufacturer’s instructions. For miRNA quantitation, reverse transcription was performed using the PrimeScript RT Reagent Kit (Takara, Dalian, China) with specific stem-loop primers. Quantitative Real-Time PCR (qRT-PCR) was performed using SYBR® Premix DimerEraser™ (Takara, Dalian, China) in a Roche LightCycler 480 II Real-Time PCR system (Roche, Basel, Switzerland). The threshold cycle value (Ct) of each product was determined and normalized against that of the internal control GAPDH or U6 (for miRNA), and the differences were compared by t-test using SPSS version 23.0, the statistical significance set at P < 0.05.

### Cell counting kit-8 assay

After treatment, the cells were seeded into 96-well plates at 2 × 10^3^ cells/well. The Cell counting kit-8 (CCK-8, Beyotime, China, C0041) reagent was injected into the wells after 0-, 12-, 24-, 48- and 72 h of culturing. Finally, the optical density at 450 nm was recorded using a microplate reader after a 2 h incubation.

### Cell cycle analysis

Cell cycle analysis was performed by using propidium iodide staining and flow cytometry. After washing once with cold PBS, cell pellets (approximately 1.0 × 10^6^ cells) were resuspended in 200 μL of cold PBS, and fixed in 4 mL of 70% ethanol overnight at −20 °C. Samples were subsequently collected by centrifugation and resuspended in 500 μL of buffer containing 40 μg/ml propidium iodide (Beyotime, China, C1052) and 100 μg/mL RNase A (Beyotime, China, C1052). All samples were incubated for 30 min at 37 °C before analysis on a CytoFLEX flow cytometer (Beckman Coulter). Data were analyzed using the CytExpert (Beckman Coulter, Version 2.0) software.

### EdU staining

EdU (5-ethynyl-2ʹ-deoxyuridine) staining was carried out using Cell-Light EdU Apoll®567 In Vitro Kit (RiboBio, Guangzhou, China, C10310). The CAL27 cells were seeded and transfected with these molecules. Then, 48 h after transfection, the cells were washed and incubated with 10 μM EdU for 30 min. Fixation and penetration of cells were performed, followed by DAPI (Thermo, USA, 62,248) staining. After washing with PBS, the plates were observed and photographed under a microscope (Olympus, Tokyo, Japan, CX41-72C02) at 200× magnification.

### Dual-luciferase reporter gene assay

Vectors, pmirGLO-PRR11 3ʹ-UTR-wt (wild-type) and pmirGLO-PRR11 3ʹ-UTR-mut (miR-26b-5p binding site mutated), for the luciferase reporter assay were generated based on the pmirGLO Dual-Luciferase miRNA Target Expression Vector (Promega, Madison, WI). The plasmids were then co-transfected with miR-26b-5p or negative control (NC) mimics into CAL27 cells using Lipofectamine 2000 according to the manufacturer’s guidelines, respectively. Relative luciferase activity was measured using the Dual-Luciferase Reporter Assay System (Promega, Madison, WI).

### RNA pulldown

Biotinylated probes complementary to PRR11 mRNA were synthesized (GenePharm, Shanghai, China) using a random probe as the control. The M-280 streptavidin magnetic beads (Sigma-Aldrich, St. Louis, MO) were coated by incubation with the probes. The cell lysates of CAL27 transfected with PRR11 overexpression vectors, with wild-type or mutated miR-26b-5p binding sites, were prepared after 48 h. Lysates were then incubated with the probe-coated beads at 4°C overnight, and the molecules interacting with PRR11 mRNA were captured after washing. The bound RNAs were subsequently purified using TRIzol and the miR-26b-5p abundance was measured by qRT-PCR.

### Western blot

The harvested cells were lysed in RIPE buffer at 4°C for 30 min and centrifuged at 15,000 × g for 15 min to obtain the protein sediment. Equal amounts of total proteins were separated by SDS-polyacrylamide gel electrophoresis (SDS–PAGE) before transferring to PVDF membranes (Millipore, Billerica, MA). Subsequently, the membrane was incubated with primary antibodies against PRR11 (Invitrogen, CA, USA, MA5-26,460, 1:2,000 dilution), cyclin D1 (Bioss, Beijing, China, bs-0623 R, 1:500 dilution), p-Rb^Ser807^ (abcam, CA, UK, ab131264, 1:1,000 dilution) and β-actin (ProteinTech, IL, USA, 66,009, 1:2,000 dilution) overnight at 4°C. After incubation with the corresponding secondary antibody (ProteinTech Group Inc., Chicago, IL, 1:6,000 dilution) for 1 h at room temperature, the signals were measured using an enhanced chemiluminescence (ECL) kit (Pierce, Rockford, IL).

## Results

This study was designed to investigate the role of the miR-26b-5p/PRR11 axis in TSCC. We studied the expression pattern of miR-26b-5p in TSCC, as well as its role in cell proliferation and cell cycle progression. Together with the prediction of the miR-26b-5p binding site within the PRR11 3ʹ-UTR, we hypothesized and verified that miR-26b-5p could suppress TSCC cell proliferation by targeting PRR11.

### miR-26b-5p was downregulated in TSCC tissues

**Figure 1.**
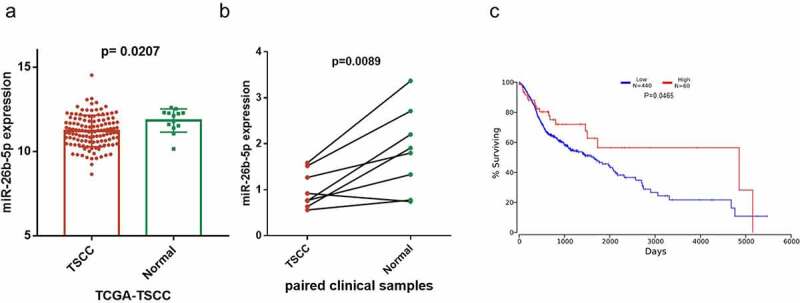
Expression and prognostic value of miR-26b-5p in TSCC. (a) the level of miR-26b-5p in TSCC tissues in TCGA dataset compared with normal tongue tissues. (b) comparison of miR-26b-5p expression in 8 paired samples collected from the TSCC patients. (c) kaplan-meier plots for overall survival (OS) in TCGA-HNSC patients, grouped by low and high expression of miR-26b-5p. P values were obtained using log-rank test

Using the data from the TCGA-TSCC subset, we found that miR-26b-5p was significantly downregulated (*p* = 0.0207) in TSCC tissues as compared with the normal tongue tissues (Figure 1(a)). To validate the results of high-throughout sequencing, the expression levels of miR-26b-5p were subsequently examined in collected tissues by qRT-PCR. Compared with the matched para-cancer tissues, the expression of miR-26b-5p was lower (*p* = 0.0089) in TSCC tissues (Figure 1(b)). Moreover, the Kaplan-Meier survival curve suggests poor OS with lower expression of miR-26b-5p than those in the high miR-26b-5p express ion group (Figure 1(c)).

### miR-26b-5p inhibits cell proliferation of CAL27

**Figure 2.**
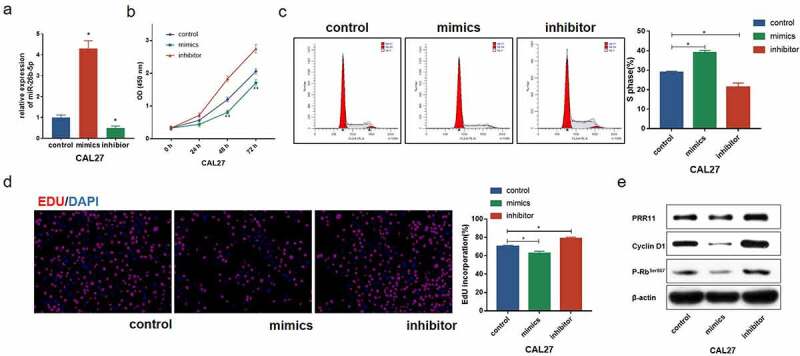
Effect of miR-26b-5p on cell cycle progression in CAL27 cells. (a) effects of miR-26b-5p mimics and inhibitor on miR-26b-5p expression in CAL27 cells. (b) the viability of each group of cells was detected by CCK-8 assay. (c) for each group, cell cycle distribution was detected by flow cytometry analysis. (d) EdU staining (red) was performed to check the proliferating cells. cell nucleus were stained with DAPI (blue). (e) levels of PRR11, cyclinD1, and p-Rb^Ser807^ were detected by western blotting analysis in each group, with β-actin as the reference protein. *, p < 0.05

Because miR-26b-5p was significantly downregulated in TSCC, we evaluated the effect of miR-26b-5p on cell proliferation of CAL27 cells. miR-26b-5p expression in CAL27 cells was regulated by transfection with miR-26b-5p mimics and inhibitors (Figure 2(a)). As indicated in Figure 2(b)), miR-26b-5p mimics inhibited the growth of CAL27 cells, whereas the inhibitor enhanced the growth of CAL27 cells. We further examined the effects of miR-26b-5p on cell cycle modulation in CAL27 cells. Compared with the control, miR-26b-5p mimics caused cycle arrest at the S-phase, and restrained CAL27 cell transit to the G2/M phase (Figure 2(c)). Further EdU staining indicated that miR-26b-5p mimics led to reductions in S phase cells, but miR-26b-5p inhibitor significantly increased the number of proliferating cells (Figure 2(d)), indicating the suppressive role of miR-26b-5p on cell proliferation. To decipher the potential mechanism of miR-26b-5p in regulating CAL27 cell proliferation, we predicted the downstream target of miR-26b-5p using the ENCORI database (http://starbase.sysu.edu.cn/), and found that there was a binding site for miR-26b-5p in the PRR11 mRNA 3ʹ-UTR. Consequently, the expression of PRR11 and cell cycle-related proteins, including CDK1 and p-Rb^Ser807^, was detected by western blotting. Our results showed that miR-26b-5p mimic decreased the protein levels of PRR11, cyclinD1 and p-Rb^Ser807^, which are the indicators of enhanced cell cycle progression, while miR-26b-5p inhibitor elevated the expression levels of these proteins (Figure 2(e)). Collectively, these findings suggest that miR-26b-5p suppresses CAL27 cell proliferation by arresting the cells at the S/G2 transition.

### PRR11 is a target of miR-26b-5p in CAL27

**Figure 3.**
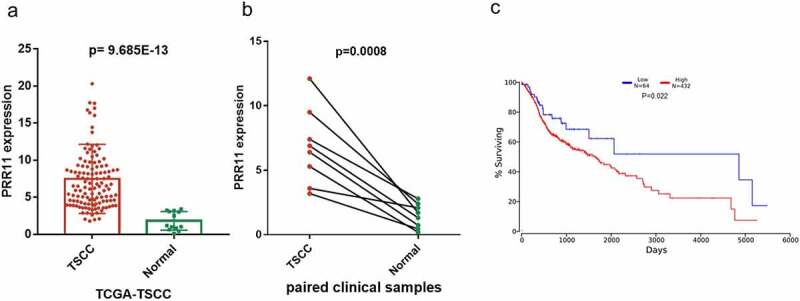
Expression and prognostic value of PRR11 in TSCC. (a) the level of PRR11 in TSCC tissues in TCGA dataset compared with normal tongue tissues. (b) comparison of PRR11 expression in 8 paired samples collected from TSCC patients. (c) kaplan-meier plots for overall survival (OS) in TCGA-HNSC patients, grouped by low and high expression of PRR11. P values were obtained using log-rank test

**Figure 4.**
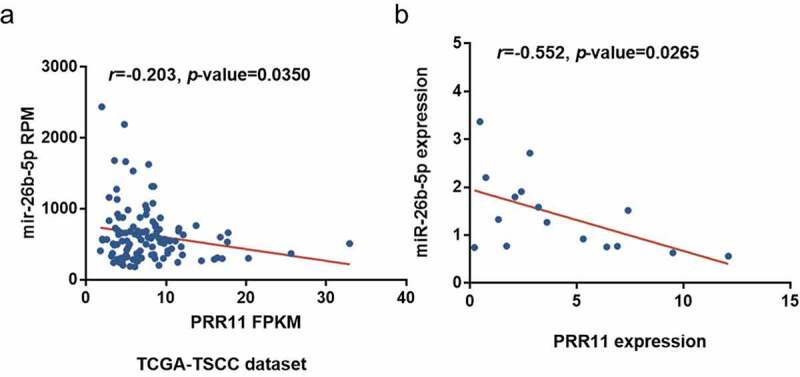
Correlation of miR-26b-5p and PRR11 expression in TSCC. pearson correlation analysis shows a negative correlation between miR-26b-5p and PRR11 mRNA level in the (a) TCGA-TSCC subset (n = 110) and (b) 8 paired samples

**Figure 5.**
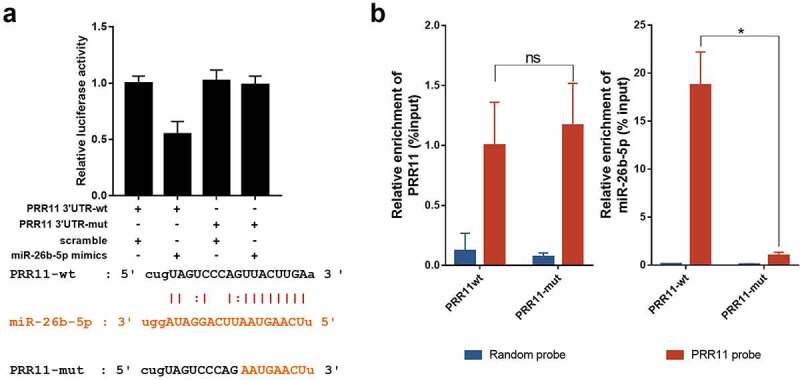
miR-26b-5p repressed PRR11 by binding to the 3ʹ-UTR of PRR11. (a) effects of miR-26b-5p on the luciferase activity of the reporter gene inserted downstream of the wildtype and mutated PRR11 3ʹ-UTR in CAL27 cells. the sequence of the miR-26b-5p binding site in the 3ʹUTR of PRR11 mRNA and its corresponding mutation were indicated. (b) RNA pulldown assay was performed in CAL27 cells transfected with wildtype and mutated PRR11, followed by qRT-PCR to detect the abundance of PRR11 and miR-26b-5p. data are presented as the mean ± standard deviation (SD). ns, not significant; *, p < 0.05

We found that PRR11 was significantly upregulated in TSCC tissues as compared with the normal tongue tissues in the TCGA-TSCC subset (Figure 3(a)) and collected paired samples (Figure 3(b)) (*p* = 0.0207 and *p* = 0.0207). Moreover, the Kaplan-Meier survival curve suggests poor OS with high expression of PRR11 compared with those in the low PRR11 expression population (Figure 3(c)). A negative correlation between the expression of miR-26b-5p and PRR11 was validated in the TCGA-TSCC subset and collected samples (Figure 4(a-b)). Luciferase reporter assays and RNA pull-down assays were also performed to further investigate the interaction between these two molecules. Transfection with miR-26b-5p mimics significantly decreased the luciferase activity of the PRR11 3ʹ-UTR wild-type reporter gene, but had no effect on that of the PRR11 3ʹ-UTR mutated reporter gene (Figure 5(a)). These results suggest that miR-26b-5p inhibits PRR11 dependent on 3ʹ-UTR binding. Furthermore, mutation of PRR11 3ʹ-UTR significantly decreased the abundance of miR-26b-5p captured by PRR11 probes (Figure 5(b), right panel), further validating the binding interaction between miR-26b-5p and PRR11.

### miR-26b-5p inhibited cell cycle progression via targeting PRR11 in CAL27

**Figure 6.**
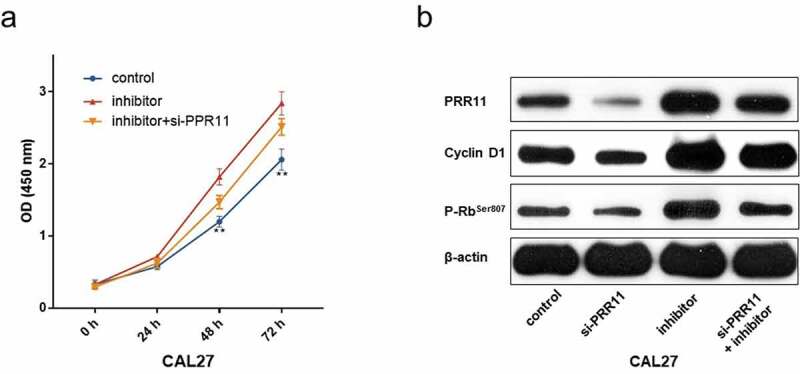
PRR11 knockdown attenuates the repression effects of miR-26b-5p on CAL27cell proliferation. (a) the viability of each group of cells was detected by CCK-8 assay. (b) western blot analysis of the impact of si-PRR11 on PRR11, cCyclinD1 and p-RbSer807 expression. data are presented as the mean ± SD

To determine the specific role of the miR-26b-5p/PRR11 axis in TSCC, we co-transfected miR-26b-5p inhibitor and si-PRR11 into CAL27 cells. The CCK-8 assay results indicated that PRR11 knockdown attenuated the effect of miR-26b-5p inhibitor on cell proliferation (Figure 6(a)). Moreover, western blotting revealed that transfection of si-PRR11 downregulated the expression of PRR11 in CAL27 cells, and weakened the facilitation effect of miR-26b-5p inhibitor on the expression of cell cycle-related genes (Figure 6(b)). These results highlight that the miR-26b-5p/PRR11 axis is involved in modulating the cell cycle progression of CAL27 cells.

## Discussion

The treatment of TSCC patients remains unsatisfactory in recent years, facilitating research on the molecular mechanisms of malignant phenotypes in TSCC. Cell cycle control provides cancer cells with a growth advantage, and can be investigated as a promising therapeutic target for TSCC treatment. In the present study, we found that the dysfunction of the miR-26b-5p/PRR11 axis were involved in the regulation of cell cycle progression. Moreover, the expression of the miR-26b-5p/PRR11 axis was significantly associated with OS in TSCC patients.

Recently, accumulating evidence has indicated that miRNAs are involved in the regulation of cell cycle progression in cancer [21,22]. However, few studies have focused on the tumorigenesis and progression of tongue cancer. In the last decade, several studies have identified miR-26b-5p to be crucial in the development and progression of cancers. As reported, miR-26b-5p is a tumor suppressor and modulator in cell cycle regulation that targets different genes, including *CCND2, PLOD2, JAG1, MAP3K9* and *KPN2* [6,8,23–25]. Notably, the ceRNA hypothesis sparked another miRNA-mediated mechanism, in which miRNA acts as the key modulator linking competing endogenous RNAs, including long non-coding RNA (lncRNA), circular RNA (circRNA), pseudogenes and protein-coding genes. For instance, circRNA_000203 exacerbates cardiac hypertrophy via the miR-26b-5p/Gata4 axis [26]. LINC00657 represses miR-26b-5p and enhances COMMD8 expression to promote NSCLC progression [27]. In addition, lncRNA HCG11 participates in the regulation of HUVEC proliferation by suppressing miR-26b-5p on QKI-5 expression [28]. These results support the idea that miR-26b-5p is implicated in the negative regulation of cell growth, which is consistent with our findings. In the current study, we identified PRR11, a promising oncogene, as a novel target of miR-26b-5p in TSCC. The expression of PRR11 is closely related to tumorigenesis, progression and poor prognosis in cancers, including lung, gastric, pancreatic, breast, esophageal and ovarian cancers [15,29–33]. PRR11 was also demonstrated to promote anti-estrogen resistance in breast cancer by amplifying the PI3K signaling pathway [34]. Additionally, PRR11 activated the Akt/mTOR autophagy signaling pathway to facilitate tumorigenesis in non-small cell lung cancer, suggesting that this gene may affect cancer cells through different signal transduction pathways. All the aforementioned results support the results of our study. To further validate the roles of the miR-26b-5p/PRR11 axis in TSCC, we performed rescue experiments, which indicated that miR-26b-5p inhibited cell cycle progression by targeting PRR11 in CAL27 cells. Consistent with our results, a previous study has indicated that PRR11 promotes TSCC cell proliferation by regulating the expression of cell cycle-related proteins, and facilitating S/G2 phase transition [35].

## Conclusions

Altogether, we have uncovered a novel miR-26b-5p/PRR11 axis and elaborated its involvement in cell cycle regulation in TSCC. Moreover, our study provides novel insights into future understanding of the molecular mechanisms of cell cycle progression in TSCC.

## Data Availability

The datasets analyzed in the current study are available from the TCGA (cancergenome.nih.gov) repository. TCGA allows researchers to download relevant data for research and publish relevant articles.
